# How to target apoptosis signaling pathways for the treatment of pediatric cancers

**DOI:** 10.3389/fonc.2013.00022

**Published:** 2013-02-14

**Authors:** Simone Fulda

**Affiliations:** Institute for Experimental Cancer Research in Pediatrics, Goethe-UniversityFrankfurt, Germany

**Keywords:** apoptosis, signaling, Bcl-2, TRAIL, IAP proteins

## Abstract

Apoptosis represents one of the most important forms of cell death in higher organisms and is typically dysregulated in human cancers, including pediatric tumors. This implies that ineffective engagement of cell death programs can contribute to tumor formation as well as tumor progression. In addition, the majority of cytotoxic therapeutic principles rely on the activation of cell death signaling pathways in cancer cells. Blockade of signaling networks that lead to cell death can therefore confer treatment resistance. A variety of genetic and epigenetic events as well as dysfunctional regulation of signaling networks have been identified as underlying causes of cell death resistance in childhood malignancies. Apoptosis pathways can be therapeutically exploited by enhancing proapoptotic signals or by neutralizing antiapoptotic programs. The challenge in the coming years will be to successfully transfer this knowledge into the development of innovative treatment approaches for children with cancer.

## INTRODUCTION

It is now well-appreciated that there are several modes of cell death in higher organisms (Lockshin and Zakeri, 2007). Among them, apoptosis represents a key cellular program which is associated with a number of characteristic biochemical features as well as morphological parameters (Lockshin and Zakeri, 2007). The efficacy of most anticancer therapies resides in their ability to engage endogenous cell death programs in cancer cells (Fulda and Debatin, 2006). This implies that a blockade of cell death pathways provides an effective means to evade the cytotoxicity of current treatment approaches. Thus, a better understanding of the molecular events that are responsible for the dysregulation of cell death signaling networks in pediatric malignancies is expected to open new opportunities for the development of innovative treatment strategies.

## APOPTOSIS SIGNALING NETWORKS

**FIGURE 1:**
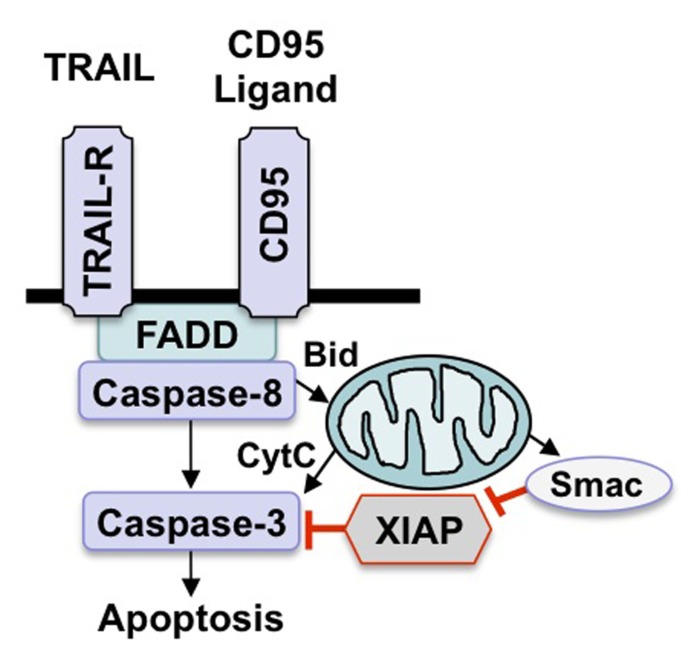
**Scheme of apoptosis pathways**. There are two key apoptosis signaling pathways, i.e., the death receptor (extrinsic) pathway and the mitochondrial (intrinsic) pathway. Stimulation of death receptors by their cognate ligands leads to activation of caspase-8, which either directly activates downstream caspases or initiates the mitochondrial pathway by cleaving Bid. Engagement of the intrinsic pathway results in the release of mitochondrial proteins such as cytochrome c or Smac into the cytosol, which in turn promotes activation of caspase-3 and apoptosis.

A core apoptotic machinery is responsible for the initiation and execution of programmed cell death by apoptosis (Fulda and Debatin, 2006). Accordingly, the death receptor (extrinsic) pathway and the mitochondrial (intrinsic) pathway represent the two principal signaling cascades that fuel into the activation of a common downstream effector route of apoptosis that eventually leads to the dismantling of the cell (**Figure 1**; Fulda and Debatin, 2006). In the executioner phase of apoptosis, a family of proteases, i.e., caspases, represent core effector molecules of apoptosis that contribute to the final steps of apoptotic cell death by cleaving key cellular substrates, for example components of the cytoskeletal apparatus and nuclear DNA (Taylor et al., 2008).

As far as the death receptor (extrinsic) pathway of apoptosis is concerned, the binding of soluble ligands of the tumor necrosis factor (TNF) family of ligands or agonistic antibodies to death receptors of the TNF receptor superfamily on the cell surface engages a signaling cascade that eventually leads to caspase activation with all subsequent subcellular events (**Figure 1**; Ashkenazi, 2008). Following ligand/receptor interaction, a multiprotein complex is formed at the plasma membrane that contains activated death receptors as well as intracellular signaling molecules that are recruited upon activation, for example FAS-associated death domain protein (FADD) and caspase-8 (Ashkenazi, 2008). This multiprotein complex is called the death-inducing signaling complex (DISC) and provides a signaling platform for activation of caspase-8 (Ashkenazi, 2008). Once activated, caspase-8 transmits the death signal by cleaving and activating downstream effector caspases such as caspase-3. Alternatively, caspase-8 can cleave other substrates besides caspases for example Bid, which results in the generation of tBid, a cleavage product with increased proapoptotic activity. tBid, in turn, translocates to mitochondrial membranes to initiate cytochrome c release. Various stress signals including DNA damage and hypoxia can engage the mitochondrial (intrinsic) pathway, leading to permeabilization of mitochondrial membranes and the release of proapoptotic proteins from the mitochondrial intermembrane space into the cytosol (**Figure 1**;Fulda et al., 2010). For example, the release of cytochrome c from the mitochondria initiates the formation of a multiprotein complex consisting of Apaf-1, cytochrome c and caspase-9 which drives caspase-9 activation in the cytosol. Alternatively, the release of Smac (second mitochondrial activator of apoptosis) fosters caspase activation and apoptosis by neutralizing Inhibitor of Apoptosis (IAP) proteins (Fulda and Vucic, 2012).

## MOLECULAR TARGETED STRATEGIES TO EXPLOIT APOPTOSIS PATHWAYS

Since the engagement of apoptosis signaling cascades can trigger cell death very efficiently and rapidly, there are also various brakes in order to ensure that accidental engagement of this cellular signaling network does not have detrimental effects on cell viability (Fulda, 2009). Accordingly, a large variety of apoptosis-inhibiting factors have been identified that prevent the execution of the full cell death program upon accidental engagement of cell death pathways. While these antiapoptotic factors constitute endogenous regulators for the fine tuning of the system, they are frequently misused by human cancers to block signaling to cell death and to evade the induction of apoptosis. For example, genetic or epigenetic events can lead to aberrant overexpression of antiapoptotic molecules or, alternatively, to silencing or inactivation of proapoptotic factors with the result of a blockade of apoptosis signaling pathways (Fulda, 2009). This implies that a better understanding of the molecular events that are responsible for the inactivation of cell death programs in human cancers can provide the basis for the development of molecular targeted therapies in order to efficiently elicit apoptotic cell death as a new therapeutic strategy. The idea to selectively target apoptosis signaling pathways in order to either directly engage cell death programs or to bypass resistance to apoptosis by neutralizing the factors that block apoptosis has in recent years already been transferred into the development of innovative treatment approaches. These concepts are currently under preclinical and clinical evaluation. On theoretical grounds, these approaches can be divided according to their primary mode of action, e.g., depending on whether they engage proapoptotic signals or antagonize antiapoptotic factors.

### STRATEGY 1: ENHANCING PROAPOPTOTIC SIGNALS

One strategy to target apoptosis signaling pathways for the treatment of cancers resides in the direct activation of proapoptotic signaling pathways (**Figure 1**). The best know example of direct engagement of apoptosis pathways is the use of death receptor ligands, which bind to the corresponding death receptors on the cell surface. Since death receptors have a direct link to apoptosis signaling pathways, this approach can directly engage the apoptotic machinery. Among the death receptors and their ligands, the TRAIL/TRAIL receptor system has gained most attention, since it preferentially triggers cell death in cancer cells compared to non-malignant cells, providing some tumor selectivity (Ashkenazi and Herbst, 2008; Humphreys and Halpern, 2008). There are four membrane-bound TRAIL receptors, two of which transmit the apoptotic signal to intracellular signaling pathways of apoptosis (Ashkenazi, 2008). By comparison, two TRAIL receptors represent decoy receptors that bind TRAIL, but do not produce a proapoptotic signal and can even antagonize TRAIL-mediated apoptosis (Ashkenazi, 2008).

In order to target the two proapoptotic TRAIL receptors, soluble TRAIL as well as antibodies directed selectively against TRAIL receptor 1 or TRAIL receptor 2 have been reported to elicit apoptosis in childhood malignancies, e.g., in neuroblastoma, rhabdomyosarcoma, Ewing’s sarcoma and leukemia (Petak et al., 2000; Van Valen et al., 2000; Kontny et al., 2001; Fulda et al., 2002a; Ehrhardt et al., 2003; Yang and Thiele, 2003; Fulda, 2008; Kang et al., 2011; Abhari et al., 2012). However, soluble TRAIL or receptor antibodies are often not sufficient as monotherapy to suppress tumor growth. For example, a fully humanized monoclonal antibody directed against TRAIL receptor 1, i.e., mapatumumab, was tested within the Pediatric Preclinical Testing Program, a National Cancer Institute initiative to identify compounds that should be prioritized for clinical evaluation in pediatric cancer (Smith et al., 2010). Limited cytotoxic effects were reported for various pediatric cancer cell lines, when mapatumumab was used in cell culture studies (Smith et al., 2010). Some antitumor activity *in vivo* was reported for mapatumumab with significant differences in event-free survival of mice treated with mapatumumab in some solid pediatric cancer models, for example osteosarcoma, neuroblastoma, and glioblastoma (Smith et al., 2010).

Based on these *in vitro* and *in vivo* studies showing that monotherapy with TRAIL ligands or TRAIL receptor antibodies are not sufficient to cause tumor regression and sustained control of tumor growth in the majority of childhood cancers, a number of different combination therapies were developed. One promising approach resides in the combination of TRAIL receptor agonists together with conventional chemotherapeutics (Van Valen et al., 2003; Komdeur et al., 2004; Muhlethaler-Mottet et al., 2004; Wang et al., 2007, 2010). The observed cooperative or synergistic interaction is considered to involve the simultaneous activation of both death receptor and mitochondrial pathways of apoptosis, resulting in enhanced activation of downstream effector caspases and, eventually, increased apoptosis. This approach of using TRAIL together with chemotherapy has been pursued in various malignancies including childhood tumors.

The evaluation of TRAIL receptor agonists against pediatric cancers is hampered by the fact that only a few studies have so far been conducted using primary tumor samples instead of established cancer cell lines. Such studies on primary tumor material are especially relevant to evaluate the antitumor activity of TRAIL receptor agonists, since it is currently unclear to what extent established cancer cell lines do in fact resemble the clinical situation. Studies testing TRAIL receptor agonists against primary tumor samples include experiments with primary neuroblastoma cells derived from children with neuroblastoma (Abhari et al., 2012). Soluble TRAIL as well as TRAIL receptor 2 agonistic antibodies were shown to trigger apoptosis in primary neuroblastoma cells, in particular in combination with molecular targeted therapeutics, i.e., Smac peptides, IAP antagonists, or proteasome inhibitors (Fulda et al., 2002b; Naumann et al., 2011; Abhari et al., 2012). Furthermore, primary acute lymphoblastic leukemia (ALL) blasts obtained from children with ALL were tested for their sensitivity toward TRAIL (Ehrhardt et al., 2003; Fakler et al., 2009). While 50% of these primary samples responded to treatment with TRAIL with apoptosis, the remaining 50% remained resistant toward TRAIL (Ehrhardt et al., 2003). Of note, some resistant samples eventually exhibited an increase in proliferation in response to TRAIL treatment compared to untreated controls, in line with the observation that TRAIL can paradoxically stimulate non-apoptotic signaling pathways proliferation in apoptosis-resistant cancers, for example by activating nuclear factor-kappaB (NF-κB; Ehrhardt et al., 2003). The first phase I clinical trial to evaluate a TRAIL receptor agonist against pediatric cancers was recently completed using the TRAIL-R2 monoclonal antibody lexatumumab in children with refractory solid tumors (**Table 2**).

### STRATEGY 2: NEUTRALIZING ANTIAPOPTOTIC PROTEINS

Another approach to exploit apoptosis signaling pathways for the treatment of pediatric cancers resides in the neutralization of antiapoptotic mechanisms that block apoptosis signaling. Depending on the status of oncogenic action of a particular tumor, this approach will either engage cell death programs or will have to be combined with additional cytotoxic principles that directly trigger cell death. Key negative regulators of apoptosis comprise the antiapoptotic proteins of the Bcl-2 family and IAP proteins.

## IAP PROTEINS AS THERAPEUTIC TARGETS IN PEDIATRIC CANCERS

Inhibitor of Apoptosis proteins comprise eight human analogs including X chromosome-linked inhibitor of apoptosis protein (XIAP), cIAP1, cIAP2, survivin, apollon, livin/melanoma-IAP (ML-IAP), neuronal apoptosis inhibitory protein (NAIP), and IAP-like protein-2 (ILP-2) (**Table 1**;Fulda and Vucic, 2012). The baculoviral IAP repeat (BIR) domain represents the unifying structural motif that is present in all IAP proteins. In addition to this BIR domain, additional functional domains of IAP proteins exist in some but not all family members. For example, the really interesting new gene (RING) domain exerts E3 ubiquitin ligase activity and is involved in ubiquitination. Despite their name, IAP proteins vary in their ability to block apoptosis. Among the IAP proteins, XIAP is considered to possess the most prominent antiapoptotic activity and inhibits caspase activation by binding to caspase-3, -7, and -9 (Eckelman et al., 2006). IAP proteins that harbor a RING domain can trigger proteasomal degradation of other IAP proteins or caspases via their E3 ligase activity (Fulda and Vucic, 2012). In addition, K63-linked ubiquitination results in modulation of signal transduction and, for example, activation of NF-κB by cellular inhibitor of apoptosis protein (cIAP) proteins.

**Table 1:** List of human IAP proteins.

Official name	Other name(s)
BIRC1	NAIP
BIRC2	cIAP1
BIRC3	cIAP2
BIRC4	XIAP
BIRC5	Survivin
BIRC6	Apollon, BRUCE
BIRC7	ML-IAP, Livin
BIRC8	ILP2, hILP2

Since IAP proteins block downstream effector pathways of apoptosis by inhibiting caspase activation and also impinge on critical cell survival pathways including NF-κB signaling, they represent promising targets for therapeutic intervention. Accordingly, a number of different approaches have been pursued over the last years to develop therapeutic strategies directed against IAP proteins. For example, Smac peptides and Smac mimetics have been developed to neutralize IAP proteins (Fulda and Vucic, 2012). These small molecules resemble the endogenous Smac protein, an endogenous antagonist of IAP proteins. Under physiological conditions, Smac resides within the mitochondrial intermembrane space. Upon the induction of apoptosis, Smac is released from the mitochondrial intermembrane space into the cytosol where it binds to IAP proteins and neutralizes them. Similar to Smac-related compounds, small-molecule IAP antagonists have been designed that similarly bind to IAP proteins and antagonize their function (Fulda and Vucic, 2012). Smac mimetics or IAP antagonists have been reported to either directly trigger apoptosis or to enhance apoptosis in response to death receptor ligands, cytotoxic chemotherapeutics or γ-irradiation in various childhood cancers, including leukemia, neuroblastoma, rhabdomyosarcoma, and glioblastoma (Fulda et al., 2002b; Fakler et al., 2009; Loeder et al., 2010; Abhari et al., 2012; Loder et al., 2012; Wagner et al., 2012). In a first proof-of-concept study in an intracranial mouse model of glioblastoma, the combined administration of Smac mimetic together with TRAIL resulted in complete eradication of glioblastoma (Fulda et al., 2002b). Similarly, IAP antagonists together with TRAIL exerted antileukemic activity in a non-obese diabetic/severe combined immunodeficient (NOD/SCID) mouse model of ALL and also acted in concert to trigger apoptosis in ALL cell lines as well as in primary blasts obtained from children with ALL (Fakler et al., 2009). Furthermore, IAP antagonists synergized together with TRAIL receptor 2 antibodies, i.e., lexatumumab, to trigger apoptotic cell death in neuroblastoma cells, including primary neuroblastoma cultures derived from patient samples (Abhari et al., 2012). Similar synergistic antitumor activity was observed for IAP antagonists together with lexatumumab against rhabdomyosarcoma. Besides TRAIL, IAP antagonists were also reported to act in concert with conventional chemotherapeutics, for example in childhood ALL (Loder et al., 2012).

Survivin represents another IAP protein that comprises one BIR domain without any additional functional domains (Altieri, 2008). Besides its function in apoptosis, survivin also plays an important role in the regulation of mitosis. As far as childhood malignancies are concerned, survivin has gained considerable attention, since it is located on 17q25, a chromosomal region that is frequently amplified in neuroblastoma (Islam et al., 2000). Accordingly, overexpression of survivin was associated with advance-stage neuroblastoma as well as poor prognosis (Adida et al., 1998; Islam et al., 2000; Azuhata et al., 2001; Ito et al., 2005; Miller et al., 2006). Besides neuroblastoma, also childhood leukemia exhibits high expression of survivin which correlated with an enhanced risk of relapse (Troeger et al., 2007). Of note, high-risk pediatric ALL also correlated with low levels of survivin-2B, since this isoform of survivin was found to promote rather than inhibit apoptosis (Troger et al., 2007). It is interesting to note that survivin has been used as a therapeutic target for immunotherapeutic strategies (Coughlin et al., 2006). For example, survivin served as tumor-associated antigene for stimulation of cytotoxic T cells. Also, a DNA vaccination based on survivin-associated peptides was designed that led to suppression of tumor growth upon prophylactic vaccination in a neuroblastoma *in vivo* mouse model (Fest et al., 2009). In children with relapsed ALL, a phase I clinical trial testing EZN-3042, an investigational agent that inhibits survivin protein expression, together with re-induction chemotherapy was recently conducted (**Table 2**).

**Table 2:** Examples of clinical trials targeting apoptosis pathways in pediatric cancers.

Agent	Target	Trial stage	Condition	Identifier #
Lexatumumab	TRAIL-R2	Phase I	Pediatric solid tumors	NCT00428272
G3139	Bcl-2	Phase I	Pediatric solid tumors	NCT00039481
EZN-3042	Survivin	Phase I	Pediatric ALL	NCT01186328

## Bcl-2 PROTEINS AS THERAPEUTIC TARGETS IN PEDIATRIC TUMORS

Proteins of the Bcl-2 family comprise both pro- and antiapoptotic members (**Table 3**) and play an important role in the regulation of the intrinsic pathway of apoptosis (Adams and Cory, 2007). Accordingly, pro- and antiapoptotic Bcl-2 proteins are critical regulators of mitochondrial outer membrane permeabilization by regulating the release of mitochondrial intermembrane proteins into the cytosol. Antiapoptotic Bcl-2 proteins such as Bcl-2, Bcl-X_L_, and Mcl-1 are often overexpressed in human cancers including childhood malignancies. Since the efficacy of chemotherapy largely depends on intact apoptosis signaling pathways, in particular mitochondria-mediated apoptosis overexpression of antiapoptotic Bcl-2 proteins has been linked to chemoresistance. Consequently, targeting of antiapoptotic Bcl-2 proteins is considered to provide a promising approach for chemosensitization of human cancers (Fulda et al., 2010). To target antiapoptotic Bcl-2 proteins, small-molecule inhibitors have been developed. For example, ABT-737 represents such a small-molecule inhibitor that binds to Bcl-2, Bcl-X_L_, and Bcl-w (Oltersdorf et al., 2005). The corresponding orally available analog ABT-263 was evaluated by the Pediatric Preclinical Testing Program (Lock et al., 2008). Interestingly, the most potent antitumor activity of ABT-263 was observed against childhood ALL both *in vitro* and *in vivo* with complete remission in 50% of cases (Lock et al., 2008). In addition, ABT-737 potentiated chemotherapy-mediated cell death, for example together with standard cytotoxic compounds used in childhood ALL including vincristin, L-asparaginase, and glycocorticoids (Kang et al., 2007). This cooperative antileukemic activity of ABT-737 together with chemotherapeutics was even observed in cases of drug resistance (Kang et al., 2007), indicating that the addition of ABT-737 may overcome some forms of chemoresistance in ALL. However, Bcl-X_L_-targeting compounds can also exert mechanism-based toxicity by inducing platelet death and subsequently thrombocytopenia (Mason et al., 2007), which may limit their clinical use in childhood cancers. Moreover, BH3 peptidomimetics that antagonize antiapoptotic Bcl-2 proteins showed antitumor activity as single agents against neuroblastoma (Goldsmith et al., 2006). Interestingly, high expression levels of Bcl-2 or Mcl-1 correlated with advanced disease in neuroblastoma (Lestini et al., 2009). Knockdown studies underscored the important role of Mcl-1 for chemoresistance, since downregulation of Mcl-1 conferred sensitivity to chemotherapeutics. *In vitro* profiling of neuroblastoma cells using BH3-domain peptides revealed that, in addition to Mcl-1, also other antiapoptotic Bcl-2 proteins can confer resistance to chemotherapy (Goldsmith et al., 2010). In addition to the small-molecule inhibitors, also Bcl-2 antisense oligonucleotides, for example G3139, showed antitumor activity in pediatric malignancies. For example, a phase I trial was conducted in children with recurrent solid malignancies together with chemotherapeutics (i.e., doxorubicin, cyclophosphamide) that demonstrated biologic activity of G3139 (Rheingold et al., 2007; **Table 2**). Together, these studies indicate that the mitochondrial pathway of apoptosis may represent a promising therapeutic target in childhood cancers.

**Table 3:** List of human Bcl-2 proteins.

Antiapoptotic	Proapoptotic
Bcl-2	Bax
Bcl-x_L_	Bak
Bcl-w	Bad
Bcl-B	Bid
Mcl-1	Bim
A1	Bik
	Bmf
	Noxa
	Puma
	Hrk

## CONCLUSION

Based on the concept that apoptotic signaling pathways are central regulators of cell death in cancers and upon cancer therapy, dysregulation of this program can lead to cancer resistance. Therefore, the identification of molecular targets that can be exploited in the design of novel anticancer strategies yielded several promising targets for therapeutic intervention. Furthermore, these strategies are already under evaluation in early clinical trials. Therefore, the knowledge on apoptosis signaling pathways and their deregulation in pediatric malignancies may open new perspectives for more effective treatment approaches for children with cancer.

## Conflict of Interest Statement

The author declares that the research was conducted in the absence of any commercial or financial relationships that could be construed as a potential conflict of interest.
